# MetaStorm: A Public Resource for Customizable Metagenomics Annotation

**DOI:** 10.1371/journal.pone.0162442

**Published:** 2016-09-15

**Authors:** Gustavo Arango-Argoty, Gargi Singh, Lenwood S. Heath, Amy Pruden, Weidong Xiao, Liqing Zhang

**Affiliations:** 1 Department of Computer Science, Virginia Tech, Blacksburg, Virginia, United States of America; 2 Department of Civil and Environmental Engineering, Virginia Tech, Blacksburg, Virginia, United States of America; 3 Department of Microbiology and Immunology, Temple University School of Medicine, Philadelphia, United States of America; 4 Department of Civil Engineering, Indian Institute of Technology Roorkee, Roorkee, Uttarakhand, India; Beijing Institute of Genomics Chinese Academy of Sciences, CHINA

## Abstract

Metagenomics is a trending research area, calling for the need to analyze large quantities of data generated from next generation DNA sequencing technologies. The need to store, retrieve, analyze, share, and visualize such data challenges current online computational systems. Interpretation and annotation of specific information is especially a challenge for metagenomic data sets derived from environmental samples, because current annotation systems only offer broad classification of microbial diversity and function. Moreover, existing resources are not configured to readily address common questions relevant to environmental systems. Here we developed a new online user-friendly metagenomic analysis server called MetaStorm (http://bench.cs.vt.edu/MetaStorm/), which facilitates customization of computational analysis for metagenomic data sets. Users can upload their own reference databases to tailor the metagenomics annotation to focus on various taxonomic and functional gene markers of interest. MetaStorm offers two major analysis pipelines: an assembly-based annotation pipeline and the standard read annotation pipeline used by existing web servers. These pipelines can be selected individually or together. Overall, MetaStorm provides enhanced interactive visualization to allow researchers to explore and manipulate taxonomy and functional annotation at various levels of resolution.

## Introduction

The field of metagenomics has arisen following the advent of next-generation DNA sequencing. Through new technologies, such as Illumina and pyrosequencing, it is now possible to directly shot-gun sequence DNA extracted from various environmental samples, without the need for cloning. Metagenomics is particularly promising for advancing the understanding of the structure and function of microbial communities residing in natural, human, and engineered environments. To date, metagenomic data sets have been obtained from different regions of the human body [1, 2, 3], seas and oceans [4, 5, 6], lakes and rivers [7, 8, 9], wastewater and drinking water treatment systems [10, 11, 12, 13], soil [14, 15], and air [16, 17]. Unlike single organismal genomic characterization, metagenomic data sets contain DNA sequences derived from hundreds or even thousands of microbial species [18, 19]. Thus, a major computational undertaking is to annotate metagenomic samples in terms of the kinds of microbes (taxonomy) and genes (functional annotation), particularly those that are present in complex environmental samples.

Various computational resources have been developed for taxonomic and functional annotation of metagenomics data sets. These resources can be classified into two main categories: 1) Web services organized as a collection of different computational resources that facilitate the storage, analysis, and retrieval of metagenomic data (e.g., MG-RAST [20] and EBI-Metagenomics [21]); 2) stand-alone programs for various aspects of metagenomic data annotation (e.g., MEGAN [22], MOCAT [23], QIIME [24], MetaPhlAn [25], MetaHIT [26], and MyTaxa [27]), which have been commonly incorporated into Web services. Generally, current services (MG-RAST and EBI-Metagenomics) annotate metagenomic samples by matching raw sequences against a fixed set of large reference sequence databases (e.g., UniProtKB [28], Clusters of Orthologous Groups of proteins (COG) [29]. This practice has two major limitations. First, there is a lack of user customization, particularly the inability to select specific sets of genes. Thus, all annotations are made with respect to the same reference databases, which may not be the most suitable depending on the hypotheses driving the research. The ability to select and focus on desired sets or subsets of reference sequences enables testing of domain-specific hypotheses. For instance, conclusions of studies of antibiotic resistance gene occurrence in the environment (e.g., [30]) can vary depending on the database selected, i.e., CARD [31], a specialized antibiotic resistance gene database, versus the full GenBank database. Second, due to short sequence length, the ability to assemble reads can be critical to identifying genes of interest and avoiding loss of information. The assembly of raw reads into longer contigs/scaffolds has proved to be more effective for annotating sequence features such as operons, transcription binding sites, chromosome organization and taxonomy [19, 32].

Here we introduce a new online metagenomic analysis server, MetaStorm, which improves available web resources, particularly for environmental samples, while maintaining a user-friendly interface. MetaStorm offers both read matching and assembly-based annotation pipelines, while also enabling customization of reference databases. This allows users to upload databases containing curated genes of interest to facilitate functional and taxonomic annotation. MetaStorm also provides enhanced visualization of annotation results, allowing the user to explore and manipulate taxonomic and functional annotations at various levels of resolution and to compare annotation for similarities and differences across multiple data samples using various graphs.

## Materials and Methods

Raw data is submitted to the MetaStorm server via a user-friendly web interface. Submitted data can remain private or be made public depending on user preference. Users are required to create an account and a profile. This profile allows them to retrieve, submit, analyze, and compare not only their own samples but also other public projects. MetaStorm stores the metagenomics samples and results into user projects which describe the features of the metagenomic experiments. If a project is made public, the raw and any associated results are free for download.

### Required data types

MetaStorm requires the user to upload raw sequences in the widely-used FASTQ format [33]. Any high-throughput DNA sequencing technology (e.g., amplicon or shotgun sequencing) is accepted. Provision of detailed metadata associated with the samples from which the DNA sequences were derived is mandatory during the submission process. Provision of metadata is critical to help users identify similar studies that are already in the MetaStorm repository for additional sample comparisons. Data is organized in a manner that facilitates retrieval. A project may contain several samples and each sample may be nested with several associated studies within it (e.g., taxonomy annotation, antibiotic resistance, or any functional annotation using both assembly and read matching pipelines). All user, sample, and project information is stored in a relational database.

### Reference database

Apart from a set of standard databases (e.g., CARD [31], UniProtKB [28], and GREENGENES [34]) (Table 1), MetaStorm also allows users to upload and use their own customized databases as reference databases. The customizability of reference databases is especially useful when researchers seek to test a hypothesis by comparison against a very specific set of sequences. Neither MG-RAST nor the EBI-metagenomics Web service allows for customized reference databases. In this way, MetaStorm enhances user control by allowing them to select reference sequences.

**Table 1:** Default reference databases provided by the MetaStorm Web service.

Database	Source	Type	#IDs	annotation
**UniProtKB**	http://www.uniprot.org/help/uniprotkb	protein	551,705	function
**CARD**	http://arpcard.mcmaster.ca/	protein	4,120	function
**ACLAME**	http://aclame.ulb.ac.be/	protein	122,154	function
**BACMET**	http://bacmet.biomedicine.gu.se/	protein	444	function
**CAZy**	http://www.cazy.org/	protein	281,237	function
**SILVA**	http://www.arb-silva.de/	nucleotide	1,756,783	taxonomy
**COG**	http://www.ncbi.nlm.nih.gov/COG/	protein	346,378	function
**GREENGENES**	http://greengenes.lbl.gov/cgi-bin/nph-index.cgi	nucleotide	1,262,986	taxonomy

### Web-based submission

**Fig 1:**
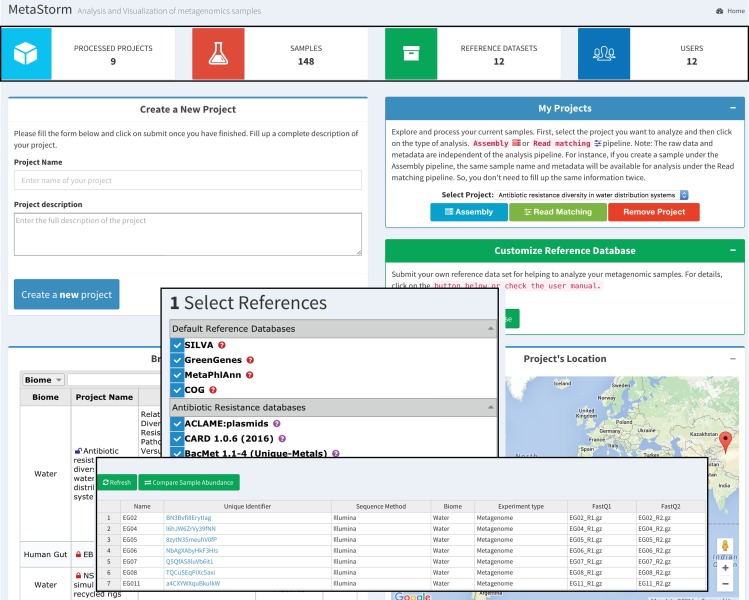
Main user interface of MetaStorm. Create a new project allows to submit a project under the user profile. My Projects grant access to the data management interface that includes: Upload raw files, add samples, remove samples, visualize individual samples and compare samples. Customize Reference Database gives access to the form for uploading a customized reference database. Browse projects allows to find samples by biome and/or location. Comparison tool allows users to compare samples from different projects. Profile allows users to modify their personal information and password.

Submission of metagenomic data is made by an interactive web interface (**Fig 1**). Users are first required to login into the MetaStorm website, select (or create) the project they wish to analyze, and select the desired method (Assembly/Read matching). Once in the project profile page, users need to insert sample information (number of samples, name of the samples, conditions, environment, and library preparation), select reference databases, upload raw FASTQ files, and finally run the annotation pipeline. To simplify the process of data submission, MetaStorm does not require external files such as Excel spreadsheets for sample description and provision of metadata (although this functionality can be easily added for future update if necessary). This interactive tool also allows users to remove samples and projects or re-run the samples with different pipelines, visualizing the results as needed.

### Analysis pipeline

**Fig 2:**
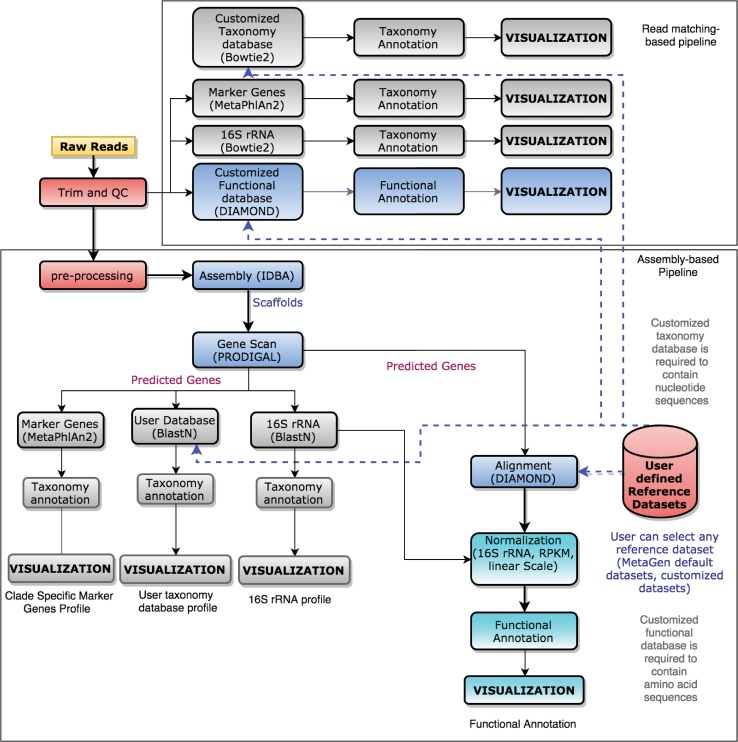
Pipelines. Overview of the computational pipelines implemented in the MetaStorm service for taxonomic and functional annotation.

Once stored in the MetaStorm server, raw reads are queued for taxonomic and functional annotations. MetaStorm incorporates two pipelines, the assembly-based pipeline and the read-matching pipeline (**Fig 2**). Selecting the appropriate pipeline depends of several parameters including: the design of the experiment, the previous knowledge about the experiment, the research hypothesis and goals. For instance, if the objective is to characterize the most abundant taxonomy in the community, the assembly pipeline may suffice [18].

#### Assembly pipeline

Through the assembly process, metagenomics reads are merged into large contiguous sequences varying in length from several hundred bases to nearly complete genomes providing much richer information relative to the raw reads [18, 19]. MetaStorm provides a fully automated assembly pipeline that allows the user to visualize, compare, and analyze the taxonomy and functional content of a sample or set of samples by matching and computing the abundance. The pipeline for assembly and gene finding is similar to the methods reported from the MetaHIT consortium [26] (mainly the metagenome assembly and gene prediction through scaffolds). This pipeline consists of the following major procedures:

1. **Quality control (QC):** reads are trimmed and filtered out by TRIMMOMATIC [35] to remove low quality sequences from the data set.
2. **Assembly:** IDBA-UD [36] is a widely used metagenome assembler that has demonstrated consistent production of high quality scaffolds [37, 38, 39]. IDBA-UD is used to assemble the QC filtered reads. MetaStorm uses the default parameters.
3. **Gene prediction:** Once a set of scaffolds are assembled, PRODIGAL [40] (metagenomics version), a microbial gene finding program, is deployed to predict genes within each scaffold.
4. **Taxonomy annotation:** Predicted genes are matched to a reference database using two alignment tools (BLAST [41] and DIAMOND [42]). Currently included are the following databases:
  - Two 16S rRNA databases (SILVA [43] and GREENGENES [34]). The 16S rRNA gene abundance is computed by first selecting the best hit (same definition as in MG-RAST representative hit [44]) to the scaffold-genes from the reference database using BLASTN [41] and then computing the number of genes that each taxa contains (E-Value<1e-10, identity >90%). Note that the taxonomy profile is computed based on the abundance of predicted genes, not the number of reads.
  - A set of marker genes processed by the MetaPhlAn2 [45] pipeline. This technique is included because whole genome sequencing samples typically contain very low 16S rRNA sequence content [26, 27, 45].
5. **Functional annotation:** Predicted genes (translated proteins from PRODIGAL) are matched to the user selected reference databases using the DIAMOND BLASTP aligner [42]. We use the representative hit strategy with an E-value<1e-10, identity>60% over the entire length [46], and minimum length of 25aa. The reference sequence databases for functional annotation depend on the user criteria. For instance, a user interested in antibiotic resistance genes may prefer to run the analysis over the CARD database [31], whereas a project related to the degradation process may use the CAZy database [47].

#### Read matching pipeline

The read matching pipeline conducts taxonomic and functional annotation of metagenomic data comparing the raw sequence reads to a reference database. This approach is also called *marker gene analysis* [18]. For taxonomy annotation, MetaStorm uses a matching scheme similar to MG-RAST and EBI-metagenomic where reads are first trimmed out and quality filtered using TRIMMOMATIC [35] and then mapped to a 16S rRNA sequence database (SILVA/GREENGENES). To speed up the read matching process, we use Bowtie2 [48], a fast and sensitive read matching tool specialized for mapping short reads to reference genomes (—local-sensitive, identity>90%, best-hit-alignment). It has proven to be particularly efficient for matching marker gene databases; MetaPhlAn2 [45] using Bowtie2 for read matching produced more accurate results than its earlier version MetaPhlAn1 [25] that uses BLAST. MetaPhlAn2 [45] which uses a set of clade specific genes is also offered by MetaStorm to estimate the taxonomic abundance. Functional annotation is made comparing the high quality reads to the reference database using the DIAMOND BLASTX [42] aligner with the representative hit approach [44] (E-value<1e-10, identity>90%, and minimum length of 25aa).

#### Sample normalization and comparison

Sample comparison consists of the analysis of relative abundance through a set of samples, allowing the user to visualize similarities and differences among samples. One of the critical aspects of sample comparison is data normalization. MetaStorm implement three different normalization techniques as follows:

1. **Scaling**: Normalize the number of matches obtained per sample, with relative abundance between 0 and 100.
2. **RPKM**: Normalize the number of matches using the Reads per Kilobase per Million Mapped Reads of each gene.
3. **Relative to 16S rRNAs**: We use the normalization concept described in [30], which defines the relative abundance as the copy of a functional gene per copy of 16S rRNA genes.

Normalizations are calculated differently for both pipelines. For the assembly-based pipeline all the computations are made in terms of number of *matched genes* whereas the read-matching pipeline normalize the samples using the number of *matched reads*.

### Visualization of taxonomic abundance

**Fig 3:**
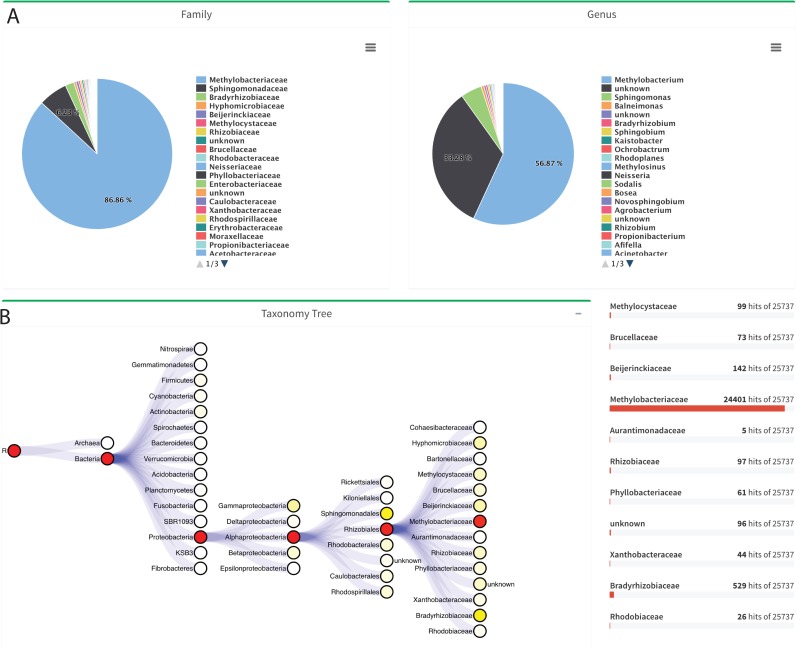
Taxonomy visualization. Taxonomy levels are shown as pie charts (only Family and Genus are shown for illustration). The interactive tree allows users to follow the path of the abundant taxas and the chart displays the selected taxonomy level. The right panel shows the hits distribution to the open node in the taxonomy tree. In this example, the families under the order *Rhizobiales* are shown in the left panel.

MetaStorm offers interactive visualization, allowing users to see in detail the main features of the sequence make-up of each sample. A taxonomic tree encodes relative abundance information of different lineages in the sample. For example, in **Fig 3**, a user interested in the relative abundance of various kinds of *Proteobacteria* will find that the genus *Achromobacter* is the most abundant. Unlike other metagenomic tools, such as MG-RAST and EBI-metagenomics, we allow interactive visualization to improve the user experience. In particular, the tree allows users to keep track of various levels of the phylogenetic hierarchy. Also, when the user clicks on any specific node (taxa), all descendants from that node will be displayed as a pie chart. The overall abundance of a taxonomy level can also be displayed as a pie chart. Node colors represent relative abundance. All visualization formats are available for the taxonomic annotation methods.

### Visualization of functional abundance

**Fig 4:**
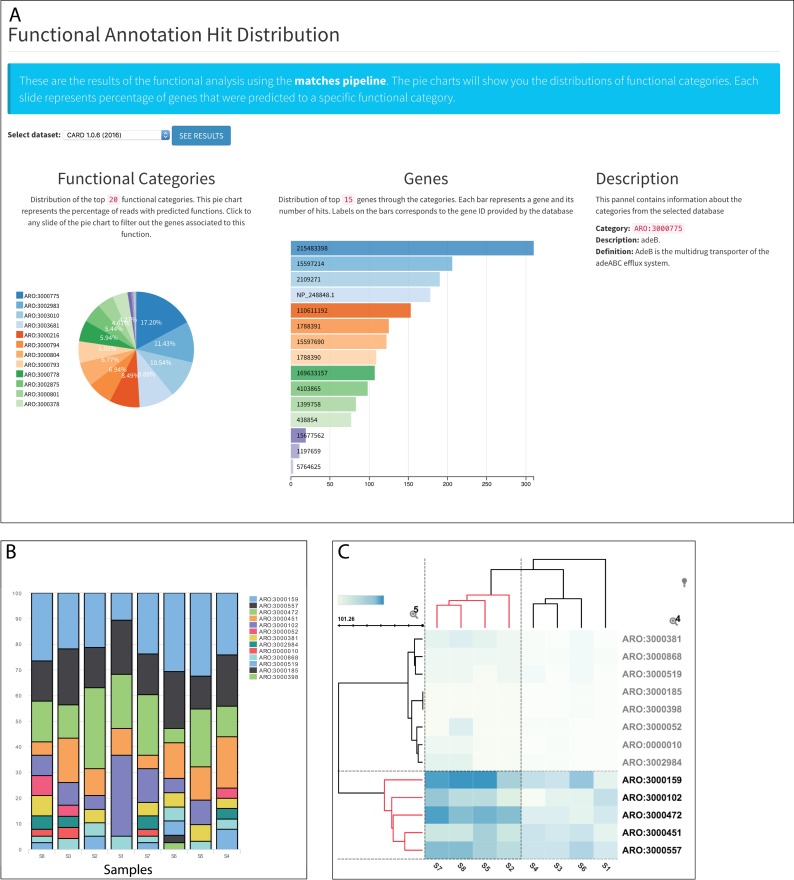
Functional and sample comparison visualization. (A) Functional annotation is depicted by a pie chart, where the user can select the database to visualize. (B) Sample comparison visualization using stacked bars for both taxonomy and function. (C) interactive heat map visualization where users can click on the branches to zoom over the related functions or taxas.

Functional relative abundance is described by a set of interactive pie charts and bar plots (**Fig 4A**) that relate functional categories with the genes involved in each category. Users can select the reference database to analyze and all the tables in text format can be downloaded. When analyzing individual samples, read/gene counts are normalized using a linear scale between 0 to 100.

### Visualization of sample comparison

Visualization techniques employed by MetaStorm include: heat maps, stacked bars, and interactive trees (taxonomy annotation). As for single sample visualization, the response tree shows relative abundance for each node (taxa) and also for each taxonomic hierarchical level, allowing a high level of specificity. This type of interactive visualization features (**Fig 4B and 4C**) are not available in other visualization tools, such as MG-RAST or EBI-Metagenomics.

### Data Access

Similar to MG-RAST and EBI-Metagenomics, all the information on a project tagged public, such as raw read files, processed files, description files, and visualization tables, are freely available through MetaStorm. From the home page, the user can access descriptions of all the recently listed (public) projects and the reference databases that other users submitted. A search tool is available for users to identify potential sets of reference sequences that can match their analysis. MetaStorm’s reference sharing capability aims to support **1**) the focus of knowledge based on user runs and **2**) the projected run time for reporting MetaStorm results. Expectedly, small customized databases will report results faster than full reference databases. A novice user can use this database for analysis and jump to the specific biological problem, thus saving the computing time. Moreover, the search tool enables users to find similar existing metagenome samples in MetaStorm (public ones) and include them for more comprehensive comparison studies. Comparison across different samples is made feasible by the normalization criteria implemented in MetaStorm. Finally, all the raw and generated files for the metagenomic analysis can be downloaded in a variety of formats by clicking on the download button of each section in the visualization page.

## Results and Discussion

Compared to other metagenomic resources, such as MG-RAST and EBI-metagenomics, MetaStorm extends the analysis and visualization of metagenomic samples by: 1) adding a fully developed assembly-based annotation pipeline, in addition to the read matching pipeline deployed by these Web servers; 2) offering a customized analysis where the user can select and upload reference databases, which enables focus on specific genes of interest as well as inter-project comparison; and 3) interactive visualization capabilities, including an interactive taxonomic tree, which permit users to interrogate and compare specific aspects of the sequence data. MetaStorm includes a wide variety of databases used for metagenomics analysis (section customizable reference database). Those databases have been used as default by several current metagenomics resources. While the assembly pipeline implemented by MetaStorm is similar to that of the MetaHIT pipeline [26], it incorporates a more meaningful relative abundance determination in which copies are normalized to 16S rRNA gene copies [30]. Normalization enables comparison across multiple metagenomics data sets, including those generated by external labs, empowering researchers to address broad. This last feature is particularly promising for the future applicability of the MetaStorm server.

## Conclusion

MetaStorm is a free and public metagenomics resource that enables a more specific user customization through various improvements of visualization, data management, and user interactivity. MetaStorm offers two main metagenomic analysis pipelines: the read matching pipeline (similar to the current web resources) and the assembly pipeline. MetaStorm, unlike any other web resources, incorporates user reference customization, which will help to streamline the annotation process when a research hypothesis requires specific and customized databases.
